# Identifying barriers to immunotherapy access for triple-negative breast cancer: A qualitative analysis of interviews with medical oncologists

**DOI:** 10.1371/journal.pone.0361021

**Published:** 2026-10-26

**Authors:** Devika R. Jutagir, Adriana Espinosa, Melissa Lopez, Maria Claros, Taisha Gomez, Jaime Gilliland, Tiffany A. Traina, Bert Petersen Jr, Francesca Gany

**Affiliations:** 1 Department of Psychiatry and Behavioral Sciences, Memorial Sloan Kettering Cancer Center, New York, New York, United States of America; 2 Department of Psychology, The City College of New York, New York, New York, United States of America; 3 Department of Medicine, Memorial Sloan Kettering Cancer Center, New York, New York, United States of America; 4 Division of Breast Surgery, SBH Health, New York, New York, United States of America; Mayo Clinic Cancer Center, UNITED STATES OF AMERICA

## Abstract

**Purpose:**

Immune checkpoint inhibitors, a type of immunotherapy, show promise in treating triple-negative breast cancer (TNBC). Although TNBC is more prevalent among Black and Hispanic women, they are less likely than non-Hispanic White women to be treated with immunotherapy. To date, no studies have explored the reasons underlying these disparities in the use of immunotherapy. This information is crucial for the development of targeted interventions that mitigate these disparities and accordingly address TNBC-related inequities among Black and Hispanic women. The aim of this study is to qualitatively identify medical oncologists’ perspectives of barriers to immunotherapy access among patients with TNBC.

**Methods:**

Using the Consolidated Framework for Implementation Research, between May 29, 2020 and September 23, 2022, we conducted semi-structured interviews until thematic saturation with 15 medical oncologists treating New York City metropolitan area patients with breast cancer. We used inductive content analysis to examine the interviews and identify themes corresponding to barriers to access to TNBC immunotherapy.

**Results:**

The following five overarching themes emerged: (1) Mixed public perceptions about immunotherapy safety and efficacy, (2) Two-tier systems of care, (3) Timely initiation of immunotherapy, (4) Lack of dissemination of best practice guidelines leads to lack of adoption of standard of care, and (5) Side effect profile impacts use and continuation of immunotherapy.

**Conclusion:**

Large-scale studies quantifying the effect of these barriers on access to TNBC immunotherapy are needed to determine cause and inform effective interventions promoting equal access to all patients with TNBC.

## Introduction

Triple-negative breast cancer (TNBC) is highly aggressive, exhibiting a 5-year overall survival rate of 77% [1], compared to a 5-year relative survival rate of 91% for all other types of breast cancer [2]. Black women have approximately 3 times the odds of receiving a TNBC diagnosis [3], and Hispanic women have roughly 22% higher odds, than non-Hispanic White women [4]. TNBC contributes to poorer outcomes among Black and Hispanic women following a breast cancer diagnosis, including higher mortality rates than non-Hispanic White women [5].

Although TNBC lacks treatment targets of traditional breast cancer treatments (e.g., estrogen receptors, progesterone receptors, HER2/neu), TNBC is characterized by a higher proportion of tumor-infiltrating lymphocytes (TILs) and elevated programmed death-ligand 1 (PD-L1) expression—biomarkers of response to immune checkpoint inhibitors (ICIs)—a type of immunotherapy [6]. ICIs are a type of cancer treatment that use the body’s innate immune system to help fight abnormal cell growth [7]. A benefit of using ICIs is the ability to stimulate the body’s immune system to target cancer cells, unlike chemotherapy or radiation, which do not distinguish between healthy versus cancer cells [8]. ICIs have redefined standard of cancer care by improving overall progression-free survival rates in many cancers, even increasing 5-year survival in cancers such as melanoma [9].

Immunotherapy, such as ICIs, have been shown to offer clinical benefit to patients diagnosed with TNBC. In 2019, atezolizumab was the first ICI approved by the United States Food and Drug Administration (FDA) to treat TNBC, although it was later withdrawn in August 2021 [10,11]. However, based on results from the KEYNOTE-355 and KEYNOTE-522 trials, pembrolizumab, which is an ICI that is offered in combination with chemotherapy, was FDA-approved in November 2020 and in July 2021 for the treatment of advanced and early-stage TNBC, respectively [12,13]. These FDA approvals have led to the recommendation of immunotherapy in combination with chemotherapy as standard of care for most patients with TNBC [14]. The addition of pembrolizumab to chemotherapy in patients with late-stage TNBC significantly improved median overall survival to 23 months compared to 16.1 months with chemotherapy alone [15]. In patients with early-stage TNBC, the 5-year overall survival was 86.6% compared to 81.7% with chemotherapy alone [16].

Although immunotherapy has the potential to improve breast cancer outcomes in all women, inequitable access to immunotherapy among Black and Hispanic women could exacerbate existing disparities in TNBC-related morbidity and mortality. Prior studies indicate that immunotherapy is less likely to be administered to patients who are Black, on Medicaid, uninsured, and/or of lower socioeconomic status [17–19]. With the advent of immunotherapy as a promising option to treat TNBC, an important first step is to evaluate barriers to and facilitators of equitable immunotherapy access. Given that medical oncologists play a crucial role in developing a patient’s treatment plan, including making the decision of whether to offer immunotherapy, and discussing the option of immunotherapy with patients, our study aimed to explore medical oncologist-reported barriers to patients with TNBC accessing immunotherapy.

## Materials and methods

### Procedure

From May 29, 2020 to September 23, 2022, medical oncologists treating patients with breast cancer from academic medical centers, community hospitals, and private hospitals, some of which were safety net hospitals, across the New York City (NYC) metropolitan area were recruited until thematic saturation [20]. Thematic saturation was achieved after 15 interviews, when no new themes emerged in the final 2 consecutive interviews [20]. With support from collaborators in breast oncology and by researching departmental websites of hospitals with oncology clinics, we created a comprehensive list of 142 medical oncologists in the NYC metropolitan area. To ensure our sample aligned with the patient population, we employed purposive sampling to recruit medical oncologists who spoke a variety of languages and who worked in diverse settings, including academic medical centers and safety-net hospitals. Selected medical oncologists received email invitations outlining the study and confirming their eligibility and interest to participate. We also used snowball sampling to recruit additional participants via referrals from previous participants. Enrolled participants completed a semi-structured in-depth interview that lasted approximately 20−45 minutes, followed by a brief demographic survey. The interviews were conducted in English via Zoom or telephone, and audio-recorded with no personal identifiers. Participants received a $50 electronic gift card as compensation for their time, consistent with recommendations for increasing physician response rates in research [21]. Participants provided verbal agreement to participate and study staff documented agreement in Research Electronic Data Capture (REDCap). Participants received a study information sheet electronically prior to participation, which included the purpose of the study and voluntary nature. This study received approval as exempt research, written consent was waived, and verbal agreement was approved by the Institutional Review Boards at Memorial Sloan Kettering Cancer Center (X20-026) and The City College of New York (2020−0367).

### Participants

Participants were eligible for this study if they met the following criteria: 1) a medical oncologist; 2) currently treating patients diagnosed with breast cancer; and 3) work in the NYC metropolitan area. Medical oncologists not meeting these criteria were excluded from the study. From our list of 142 medical oncologists, 28 were selected and emailed. Among them, 2 had invalid email addresses, and 11 did not respond. Fifteen medical oncologists across 11 institutions in the NYC metropolitan area participated in this study.

### Semi-structured interview

A multidisciplinary team with methodological and subject matter expertise (D.R.J, A.E., and F.G.) and a qualitative methods specialist with a master’s degree in anthropology created a semi-structured in-depth interview guide based on the Consolidated Framework for Implementation Research (CFIR), a widely used implementation science framework [22]. CFIR guided domains assessed in the in-depth interviews and survey included: innovation characteristics (e.g., cost, side effect monitoring complexity), individual characteristics (e.g., clinician immunotherapy knowledge), inner setting characteristics (e.g., limitations to hospital resources), outer setting characteristics [e.g., National Cancer Institute (NCI) designation, guidelines], and implementation process (e.g., PD-L1 biomarker testing workflows). We explored these domains through interview questions encompassing 7 areas: 1) clinician awareness of and knowledge about immunotherapy guidelines; 2) Institutional logistics; 3) Support for monitoring immunotherapy side effects; 4) Clinician comfort/training/experience prescribing immunotherapy; 5) Attitudes about immunotherapy; 6) Concerns about cost/reimbursement; and 7) Bias and discrimination. The interview guide was iteratively edited throughout data collection to reflect updates in immunotherapy indication and emerging concepts to probe in future interviews. All interview sessions were conducted by a female, English-speaking clinical psychologist specializing in psycho-oncology and cancer disparities (D.R.J.), and a female, bilingual English- and Spanish-speaking bachelor’s level Clinical Research Coordinator (M.L.), both trained by a qualitative methods specialist.

### Demographic survey

After the interviews, participants also responded to a 28-question, 10-minute survey assessing demographic (9 questions, e.g., “What is your age?”) and occupational characteristics (19 questions, e.g., “Are you affiliated with an NCI-Designated Comprehensive Cancer Center?”).

### Data analysis

The audio-recorded interviews were transcribed verbatim by trained research staff and checked for accuracy by the clinical psychologist (D.R.J.). A multidisciplinary team of 5 female coders—a doctoral-level Applied Health Researcher (A.E.), a doctoral-level clinical psychologist specializing in cancer disparities and psycho-oncology (D.R.J.), a medical student (M.L.), a bachelor’s-level Community Outreach Coordinator (T.G.), and a graduate-level in public health Research Project Associate (M.C.)— were involved in the coding and analysis.

We first conducted open coding to identify key concepts and codes. Then, four coders (A.E., D.R.J., M.L., and T.G.) met to reach consensus and develop a codebook based on constructs of interest. Each transcript was coded independently by two coders using the codebook. Consensus meetings were held to finalize coding. Once finalized, we merged all individually coded transcripts into a master file. We used NVivo version 14 to generate quote reports. To generate the themes, three coders (A.E., D.R.J., and M.C.) analyzed the reports inductively and met weekly to reach consensus. The Consolidated Criteria for Reporting Qualitative Research (COREQ) guided the reporting of findings [23].

We descriptively analyzed the demographic data from the clinician survey.

## Results

### Demographics

Table 1 presents the characteristics of the sample. Participants were primarily female (93%), and they identified as White (67%), Asian (27%), and Hispanic (20%) and spoke multiple languages in addition to English, with Spanish being the most common (53%). Under half of the participants had been in practice 20 years or more (40%), over half worked in safety-net hospitals (60%), and approximately half worked in NCI-Designated Comprehensive Cancer Centers (47%). Approximately one third (33%) exclusively treated patients with breast cancer, while more than half (67%) also treated patients with other types of cancer.

**Table 1:** Characteristics of Medical Oncologists Interviewed (N = 15).

Characteristic	n (%)
**Demographics**	
Age (years)	
Mean (range)	50 (35–67)
30-39	4 (27)
40-49	2 (13)
50-59	6 (40)
60-69	3 (20)
Sex	
Female	14 (93)
Male	1 (7)
Race	
White	10 (67)
Asian	4 (27)
Other	1 (7)
Ethnicity	
Hispanic	3 (20)
Not Hispanic	12 (80)
Languages spoken^†^	
English only	3 (20)
Spanish	8 (53)
Other languages	11 (73)
**Practice setting**	
Hospital type	
Academic medical center	10 (67)
Community hospital	4 (27)
Private hospital	1 (7)
Safety-net hospital	
No	6 (40)
Yes	9 (60)
NCI-Designated Comprehensive Cancer Center	
No	8 (53)
Yes	7 (47)
NCI-Designated Cancer Center	
No	11 (73)
Yes	4 (27)
**Years in practice**	
<10	3 (20)
10 to <20	5 (33)
≥20	6 (40)
Unknown	1 (7)
**Types of cancer treated**	
Breast	5 (33)
Breast and gynecological	3 (20)
Breast and other	7 (47)

Abbreviations: NCI, National Cancer Institute.

†Languages spoken by medical oncologist does not sum to 15 as many spoke more than one language.

All percentages may not sum to 100 due to rounding.

### Themes

Interviews with medical oncologists regarding barriers to accessing immunotherapy yielded 15 key concepts that were group into five themes: 1) Mixed public perceptions about immunotherapy safety and efficacy; 2) Two-tier systems of care; 3) Timely initiation of immunotherapy; 4) Lack of dissemination of best practice guidelines leads to lack of adoption of standard of care; and 5) Side effect profile impacts use and continuation of immunotherapy (Table 2). Our thematic constructs are illustrated in a conceptual figure representing both patient- and physician-level, and practice environment barriers reported to influence access to immunotherapy (Fig 1).

**Table 2:** Main Themes and Key Concepts Reported by Medical Oncologists.

Theme	Key Concepts	Example Quotations	Code
1. Mixed public perceptions about immunotherapy safety and efficacy			
	As general knowledge of immunotherapy improved, patient expectations better aligned with its potential outcomes	*“In the past a lot of patients felt that immunotherapy was this kind of panacea, that it was going to cure everyone of their breast cancer with no side effects at all because it wasn’t chemo… But I feel like there’s a much better understanding in the public that this is just another kind of treatment that can be used to treat different kinds of cancer and that it’s not necessarily going to be this magic bullet.”* (**ID RD016**: Breast oncologist, academic medical center, 12 years in practice)	Patient awareness & knowledge of immune checkpoint inhibitors Patients’ trust in the medical system Patients’ attitude toward immune checkpoint inhibitors
	Concerns that patients’ perception of immunotherapy as experimental influenced their decisions	*“But the other thing that I think does come up when we’re talking about underserved populations is immunotherapy, while it’s exciting when things are new, there are some people that say ‘wow new is better and new has got to be great’ and there’s some people that say, ‘Hmm new, is this Tuskegee experiment?’”* (**ID RD001**: General oncologist, academic medical center, 10 years in practice)	
2. Two-tier systems of care			
	Lack of language-concordant care extends visit durations, leading to rushed and incomplete discussions about immunotherapy for patients with limited English proficiency	*“… using the interpreter lengthens the visit time, sometimes it can be kind of more rushed, or things that are missed in translation. I think it’s a more challenging conversation if English is not the first language...”* (**ID RD053**: Breast & gynecologic oncologist, community hospital, 3.5 years in practice)	Patient access to language concordant care Medical oncologist suggestions to improve systemic barriers to care Patients’ socioeconomic status/demographics affecting care
	Due to limited resources for high-cost treatments, public hospitals are unable to provide the same level of care as affiliated private hospitals	*“We have a relationship with a New York City public hospital where many of our fellows and some of our attendings also work in the breast cancer clinic there … patients often are from lower socioeconomic groups and there may be a higher incidence of African-American or other groups of individuals I think and certainly uninsured people. So, I think for those individuals it is more difficult to get anything that’s expensive … because the costs are prohibitive and I’m not sure that the public health care system can take on those expenses.”* (**ID RD013**: Breast oncologist, academic medical center, unknown years in practice)	
	Patients with Medicaid are likely to be seen by trainees instead of attending physicians	*“Maybe not all physicians are taking Medicaid and it’s possible that in some institutions a Medicaid patient is treated in a fellows clinic and not by an attending and might not have someone whose managing their case that’s as up to date about the specific new therapies in breast cancer.”* (**ID RD035**: Breast & gynecologic oncologist, academic medical center, 25 years in practice)	
3. Timely initiation of immunotherapy			
	Delays to PD-L1 testing workflows	*“At the very beginning, when we were just learning about PD-L1, it was pretty cumbersome because we had these pathology results and I had to basically case-by-case discuss and request additional molecular testing for my patients for specific disease status … I still have some delays sometimes.”* (**ID RD028**: General oncologist, community hospital, 14 years in practice)	Institutional logistics and resources for PD-L1 testing Resources for immune checkpoint inhibitor implementation Treatment delays Staffing for immune checkpoint inhibitor implementation
	Variability of time to prior authorization approvals differed across Medicaid plans	*“So, for example [Medicaid plan 1]: I just had a patient approved for pembrolizumab for lung cancer and that happened within 48 hours, but [Medicaid plan 2] for example can take up to a week or longer for approval. It just really depends on which managed Medicaid plan the patient has … Unfortunately it causes a delay in treatment ‘cause we can’t start anything … So, in some cases like I have started different treatments because of the delay.”* (**ID RD002**: General oncologist, academic medical center, 2 years in practice)	
4. Lack of dissemination of best practice guidelines leads to lack of adoption of standard of care			
	Lack of standardized continuing medical education on immunotherapy for medical oncologists	*“There are government required things that you have to do to renew your medical license, but to the best of my knowledge nobody says you absolutely have to do your continuing education in immunotherapy.”* (**ID RD001**: General oncologist, academic medical center, 10 years in practice)	Medical oncologist education/training in immune checkpoint inhibitors Resources used for immune checkpoint inhibitor information Medical oncologist experience using immune checkpoint inhibitor Medical oncologist suggestions to improve systemic barriers to care Need for immune checkpoint inhibitor training of primary care providers and ancillary care providers
	Medical oncologists familiar with immunotherapy from other cancer types, often in community hospitals, struggled to stay informed about new approvals	*“Well, the immunotherapy has been used in lung cancer earlier. So, I had baseline experience from lung.”* (**ID RD001**: General oncologist, academic medical center, 10 years in practice)	
		*“Yeah in the community oncologists I think that is a big challenge, to keep up with things … We have them [fellows and residents] read the new articles, come back and talk about it and we have a discussion. So I think that’s how we try to keep up. But yes, it is definitely a challenge and given that we are extremely clinically relatively busy and short staffed, it’s becoming a problem … ”* (**ID RD024**: General oncologist, community hospital, 4 years in practice)	
	Need for formal training or continuing education on managing immune-related side effects for healthcare professionals not specialized in oncology	*“I think other than just the spread of the knowledge to other providers—the primary care, emergency room physicians—because patients do end up to different providers who don’t give these treatments, and they are not aware, and sometimes if the patient has side effects, they do not think that it could be immune therapy-related and that side effect needs a specific management …”* (**ID RD054**: General oncologist, community hospital, 10 years in practice)	
5. Side effect profile impacts use and continuation of immunotherapy			
	Concerns that patients’ reporting of side effects is influenced by their normalization of physical discomfort, and by psychological symptoms	*“A lot of them have tolerated a lot of physical suffering already and they’re used to just sitting it out. It’s not that they don’t want to report it [side effects], but they don’t want to trouble you, or they think it’s normal …”* (**ID RD001**: General oncologist, academic medical center, 10 years in practice)	Side effect management Medical oncologist perceptions of immune checkpoint inhibitor toxicity and side effects Comorbidities/contraindications affecting immune checkpoint inhibitor use
		*“Yeah, I mean the problem with her is that she has severe history of anxiety. So, it was hard to tell was her reporting accurate on the diarrhea … And also, she was not compliant with our recommendations of anti-diarrheal …”* (**ID RD017**: Breast oncologist, academic medical center, 22 years in practice)	
	Challenges differentiating between chemotherapy versus immunotherapy side effects in chemo-immunotherapy regimens	*“Well, she developed this very unrelenting bone pain that I couldn’t attribute to anything other than... it was hard to figure out what was exactly causing it but I had never seen that with carboplatin so it might have been related to the medication, Keytruda. I’m not sure.”* (**ID RD036**: Breast oncologist, academic medical center, 20 years in practice)	
	Chemo-immunotherapy side effects can exacerbate symptoms from comorbidities and autoimmune diseases, preventing immunotherapy use	*“Yeah, so she was an African American woman with metastatic triple negative breast cancer who had been exposed to taxane therapy and she had peripheral neuropathy that was very severe, and that was complicated also by her uncontrolled diabetes … And unfortunately, because of her severe neuropathy, I couldn’t give it [chemo-immunotherapy regimen].”* (**ID RD002**: General oncologist, academic medical center, 2 years in practice)	
		*“So, there are some autoimmune diseases that can flare in the context of immunotherapy, and so in those cases we would sort of approach with caution. I’m not aware of a strict contraindication, and I might be wrong about that … The problem is if a patient who is on steroids to control like an autoimmune disease, or another condition, the steroids can interfere with the effectiveness of the immunotherapy. You also wouldn’t want the immunotherapy to cause a serious flare of a significant autoimmune disease. So, this doesn’t necessarily apply to every single autoimmune disease … ”* (**ID RD016**: Breast oncologist, academic medical center, 12 years in practice)	

**Fig 1:**
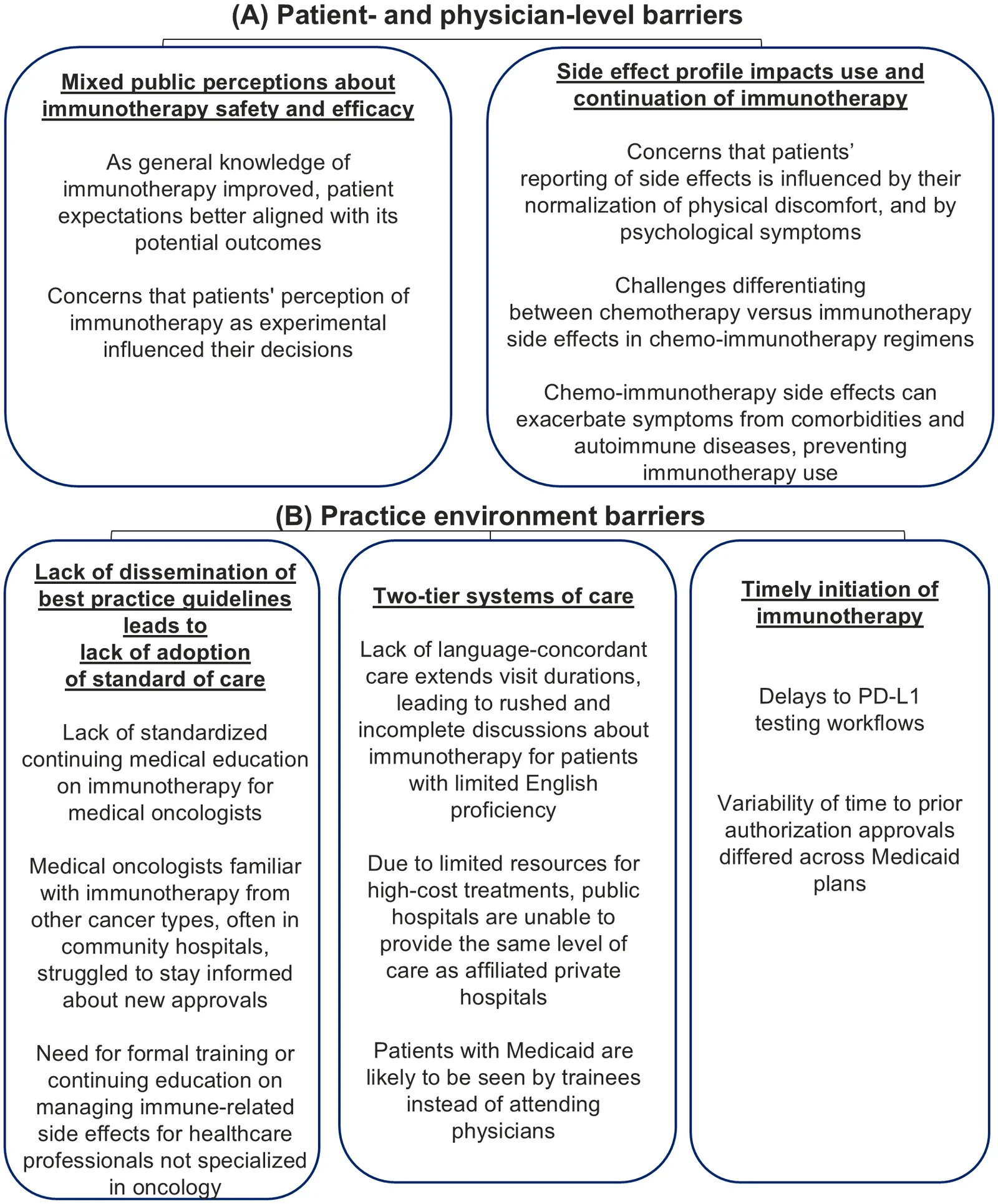
Medical oncologist-reported barriers to patients with TNBC accessing immunotherapy. A) Themes reported at patient and physician levels. B) Themes reported at the practice environment level.

#### Theme 1: Mixed public perceptions about immunotherapy safety and efficacy.

Medical oncologists noted that patients have mixed beliefs about immunotherapy’s safety and efficacy. While some patients initially perceived immunotherapy as a “panacea,” as general knowledge of immunotherapy improved, subsequent patients recognized it may not work for everyone. Medical oncologists described that perceptions of immunotherapy have shifted over time to more accurately incorporate the degree of potential clinical benefit and side effects. However, they reported that the history of experimentation without consent on underserved populations led some patients to perceive immunotherapy as “experimental” and influenced their decision to consider the treatment.

#### Theme 2: Two-tier systems of care.

Medical oncologists also noted that patients may receive varied levels of care depending on their English proficiency, treatment hospital, and insurance type, creating two-tier systems of care.

They reported that lack of language-concordant care extended visit durations, leading to rushed and incomplete discussions about immunotherapy for patients with limited English proficiency. They expressed that limited time or resources in underfunded institutions hindered their ability to fully explain immunotherapy as a treatment option to patients and confirm their understanding. One community oncologist noted that interpreter services sometimes led to information being lost in translation when treating patients who prefer a language other than English.

In cases where the same medical oncologist treated patients at both a private cancer center and its affiliated public hospital, they described that the care they provided was limited at the public hospital due to prohibitive costs for expensive treatments, which could apply to immunotherapy. Furthermore, medical oncologists reported patients insured by Medicaid may be cared for by a trainee, such as a fellow, rather than by an attending physician, regardless of the hospital they are treated in.

#### Theme 3: Timely initiation of immunotherapy.

Medical oncologists highlighted delays in starting immunotherapy, which they attributed to unstandardized workflows for testing predictive biomarkers for immunotherapy efficacy (e.g., PD-L1). They reported that the cost of maintaining onsite PD-L1 testing could be prohibitive. At safety-net hospitals, community oncologists reported that when PD-L1 tests were sent to external laboratories or institutions for analysis, it led to delays in timely initiation of treatment.

Additionally, medical oncologists shared that variability in prior authorization approval timelines differed across Medicaid plans, impacting timely initiation of immunotherapy and, in some cases, changes in the treatment regimen.

#### Theme 4: Lack of dissemination of best practice guidelines leads to lack of adoption of standard of care.

Medical oncologists further reported that the absence of continuing medical education (CME) requirements, lack of awareness of the latest treatment approvals, and limited formal training in immunotherapy create barriers to understanding and adopting immunotherapy according to standard of care. Although completing CME courses is required to renew their medical licenses, there are no CME requirements mandated for immunotherapy.

Medical oncologists who treated multiple cancer types, a model of care particularly seen in community hospitals, reported that their familiarity with immunotherapy from treating patients with other cancers prior to the TNBC indication may facilitate its adoption for the treatment of TNBC. On the other hand, these oncologists reported difficulties staying informed about new immunotherapy approvals across all cancer types, including those for TNBC, hindering implementation of immunotherapy.

They also stated formal training was necessary among clinicians across multidisciplinary specialties. They noted that immune-related side effects are not often recognized among patients evaluated in non-oncology settings (e.g., primary care, emergency room).

#### Theme 5: Side effect profile impacts use and continuation of immunotherapy.

Medical oncologists reported reluctance to prescribe immunotherapy when lacking confidence in patients’ ability to reliability report side effects. Reassurance about potential to report side effects was shaped by patient engagement in care. They highlighted that patients on immunotherapy face a unique spectrum of side effects, many of which may go underreported. Some oncologists expressed that patients, particularly those from underserved medical populations, may struggle to effectively communicate side effects because they have normalized physical discomfort or are concerned with burdening their oncologists. Additionally, they noted that a patient’s emotional state, particularly anxiety, can negatively impact accuracy in the reporting of side effects during treatment.

Furthermore, medical oncologists described challenges differentiating between chemotherapy versus immunotherapy side effects in the context of chemo-immunotherapy regimens. They reported that the combination of chemotherapy and immunotherapy approved for TNBC makes it difficult to attribute side effects to immunotherapy, complicating the effective management of side effects. Some medical oncologists also expressed that pre-existing comorbidities, such as neuropathy from diabetes, present challenges for prescribing the chemo-immunotherapy regimen approved for TNBC due to concerns of increased toxicity and side effects. The presence of autoimmune diseases, some of which are more prevalent in minoritized populations, presented similar challenges.

## Discussion

This qualitative study is the first to explore themes underlying the use of immunotherapy for TNBC from the perspective of medical oncologists. This study was guided by CFIR, which is a widely used implementation science framework. Guided by CFIR, this study identified multiple barriers to immunotherapy access that represent modifiable determinants of clinical practice. Thematic constructs spanned key CFIR domains, including both patient- and physician-level, and practice environment characteristics reported to influence immunotherapy access (Fig 1). Our findings align with current challenges identified within the field of immunotherapy [24–27].

Study findings highlight the importance of education programs for patients to address several of the barriers identified, for example, belief that immunotherapy is experimental, the risks and benefits of immunotherapy, and differences between immunotherapy versus chemotherapy. Patient education programs can teach patients to recognize and follow protocols for reporting immune-related side effects [24]. Educating patients can help them engage in shared decision-making [28] and thereby address their physician’s hesitancy to prescribe immunotherapy. Additionally, collaborations between medical oncologists and community organizations can enhance community outreach to the public about immunotherapy. Such partnerships can help ensure that patient advocates are up to date and can accurately inform patients and the public about immunotherapy.

Regarding the possible impact of two-tier systems of care on access to immunotherapy, federal policy could mirror state-level policies that have expanded access to cancer centers that offer highly specialized care, with the purpose of increasing access to immunotherapy. For example, in January 2023, New York State mandated that Medicaid plans cannot refuse coverage of treatment at NCI-Designated Comprehensive Cancer Centers [29]. NCI-Designated Comprehensive Cancer Centers invest significant resources in research, physicians, and facilities to improve approaches to cancer treatment [30]. Consequently, these centers may be more likely to have on-site PD-L1 testing, which can minimize treatment initiation delays, and increase knowledge of immunotherapy among oncologists. Other states, including California, have passed similar legislation [31], and this type of legislation can be expanded at the federal level.

Delays in access to immunotherapy could also be addressed through policy via standards set by Centers for Medicare and Medicaid Services (CMS). Given that insurance approvals typically follow standards set by CMS, streamlining the prior authorization review process can prevent delays in initiating immunotherapy. In January 2024, CMS finalized a ruling to improve the prior authorization process by requiring Medicare Advantage and Medicaid managed care plans to provide decisions within seven calendar days for standard requests and 72 hours for expedited requests. This change will go into effect in January 2026 [32]. Communication of these new prior authorization standards is essential for ensuring that medical oncologists and patients are well-informed. Delays starting immunotherapy can also be minimized by developing internal hospital procedures to hasten implementation of internal PD-L1 testing protocols when tests must be sent out externally.

To address lack of dissemination of immunotherapy best practices, professional organizations and state boards could mandate continuing education programs needed to disseminate essential information about immunotherapies like ICIs. Multidisciplinary educational programs are essential to navigate practical challenges with immunotherapy and provide evidence-based recommendations to manage immune-related side effects and inform clinical decision-making [25]. These programs can help ensure that medical oncologists are aware of current indications, and that they and other types of clinicians are able to recognize and address immune-related adverse events. While clinical guidelines about the use of immunotherapy exist in the oncology setting [33], the development of such guidelines for physicians in non-oncology settings is emerging [26]. Requirements for continuing medical education about immunotherapy may be warranted, given that clinicians who were educated before ICIs were approved for use in cancer have often not received formal training about immunotherapy and managing side effects.

Current clinical guidelines recommend immunotherapy in combination with chemotherapy as first-line treatment for most patients with TNBC [14], making it difficult to recognize immunotherapy-related, versus chemotherapy-related, side effects [27]. The strategies stated above to educate patients and clinicians about immunotherapy side effects could help address the potential issue that the ICI side effects profile limits immunotherapy access. Efforts must also be made to connect patients with psychosocial care to address psychiatric symptoms, such as anxiety, that affect the ability to accurately report or tolerate side effects. The National Comprehensive Cancer Network Distress Management Guidelines [34] recommend standards for screening patients for psychosocial distress and referring them for care. There is also a need to develop low-resource, low-intensity interventions to reduce distress among patients in cancer clinics and maximize onsite capacity to provide psychosocial care.

### Strengths and limitations

A notable strength of the study is that our sample was racially, ethnically, and linguistically diverse and worked across 11 academic medical centers and community hospitals, most of which were considered safety-net hospitals. Our sample’s demographic distribution generally reflected the national medical oncology workforce. Our findings are consistent with the race distribution of medical oncologists as reported in 2020 [35], although Hispanic participants were overrepresented in our sample (20%). Based on data collected in 2016 [36], our participants were slightly older than national demographics, with 60% older than age 50 years, versus 50% of the national medical oncology workforce over age 50. Compared with national demographics [36], our sample was more female (93%) compared with 30% of the national workforce, which aligns with our focus on breast oncologists, who may be more likely to be female. Another strength is that our study also adhered to COREQ guidelines for rigorous qualitative design, including a multi-disciplinary team of investigators.

However, there are methodological limitations that should be considered when interpreting our findings. Since recruitment was based in the NYC metropolitan area, the barriers identified in our themes may differ from those in other healthcare systems or geographic areas. Unlike in other geographic areas, patients in NYC may benefit from regional healthcare characteristics, insurance processes, and resources, such as Medicaid expansion, safety-net structures like emergency Medicaid, and extensive public transit services. Additionally, the transferability of our findings may be impacted by self-selection bias, as medical oncologists who agreed to participate could differ substantially from those who did not. Selection bias may have been introduced by email-based recruitment given that many medical oncologists from our initial list did not have email addresses available via public records. Whenever possible, we used alternative outreach methods to obtain email addresses via phone calls, LinkedIn, and in-person methods. Some of the early interviews for this study were collected during the period in which atezolizumab was FDA-approved for treatment of TNBC, whereas the TNBC indication was withdrawn in August 2021. However, consistency of themes during the period of atezolizumab approval with themes from interviews collected later after the additional approval of pembrolizumab for TNBC indicate that this change was not a major source of bias. It is possible that the medical oncologists provided socially desirable responses, potentially underreporting some barriers to immunotherapy use in TNBC (e.g., bias); future experimental designs can assess whether bias influences clinician decision-making regarding immunotherapy prescription. Given our small sample size, we were unable to analyze whether themes differed based on clinician characteristics such as race or ethnicity. Although none of the team members engaged in the coding of interviews were physicians, three physicians from different specialties reviewed the results (T.A.T., B.P., F.G.). Finally, this study is limited by its focus on physician-reported barriers, underscoring the importance of future integration of both patient and physician perspectives to more holistically characterize multilevel barriers to immunotherapy access.

### Future directions

Given the exploratory nature of this qualitative study, an important next step is to quantitatively examine these barriers and assess their associations with measurable outcomes related to immunotherapy use. For example, barriers can be categorized according to CFIR, and then validated items and measures can be identified to measure these constructs [37] in larger survey studies. Such work is essential to inform targeted interventions, such as patient navigation programs, that can increase immunotherapy access. Immunotherapy implementation could be incorporated into these interventions via categorizing barriers documented here according to CFIR constructs, then mapping these constructs to implementation strategies [38,39]. We are currently piloting a randomized controlled trial to evaluate whether a navigation program, compared to customary care, increases immunotherapy uptake.

## References

[1] Triple-Negative Breast Cancer. American Cancer Society. https://www.cancer.org/cancer/breast-cancer/about/types-of-breast-cancer/triple-negative.html. Accessed 2025 February 7.

[2] Breast Cancer Facts & Stats. https://www.nationalbreastcancer.org/breast-cancer-facts/. 2025. Accessed 2025 February 10.

[3] McCarthy AM, Friebel-Klingner T, Ehsan S, He W, Welch M, Chen J, et al. Relationship of established risk factors with breast cancer subtypes. Cancer Med. 2021;10(18):6456–67. doi: 10.1002/cam4.4158 34464510 PMC8446564

[4] Scott LC, Mobley LR, Kuo TM, Il’yasova D. Update on triple-negative breast cancer disparities for the United States: A population-based study from the United States Cancer Statistics database, 2010 through 2014. Cancer. 2019;125(19):3412–7. doi: 10.1002/cncr.32207 PMC6744285 31282032

[5] Yedjou CG, Tchounwou PB, Payton M, Miele L, Fonseca DD, Lowe L, et al. Assessing the Racial and ethnic disparities in breast cancer mortality in the United States. Int J Environ Res Public Health. 2017;14(5):486. doi: 10.3390/ijerph14050486 28475137 PMC5451937

[6] Abdou Y, Goudarzi A, Yu JX, Upadhaya S, Vincent B, Carey LA. Immunotherapy in triple negative breast cancer: Beyond checkpoint inhibitors. NPJ Breast Cancer. 2022;8(1):121. doi: 10.1038/s41523-022-00486-y 36351947 PMC9646259

[7] Immunotherapy to Treat Cancer. National Cancer Institute. https://www.cancer.gov/about-cancer/treatment/types/immunotherapy. 2019. Accessed 2025 February 7.

[8] Day D, Monjazeb AM, Sharon E, Ivy SP, Rubin EH, Rosner GL, et al. From famine to feast: Developing early-phase combination immunotherapy trials wisely. Clin Cancer Res. 2017;23(17):4980–91. doi: 10.1158/1078-0432.CCR-16-3064 28864726 PMC5736967

[9] Larkin J, Chiarion-Sileni V, Gonzalez R, Grob J-J, Rutkowski P, Lao CD, et al. Five-year survival with combined nivolumab and ipilimumab in advanced melanoma. N Engl J Med. 2019;381(16):1535–46. doi: 10.1056/NEJMoa1910836 31562797

[10] FDA approves atezolizumab for PD-L1 positive unresectable locally advanced or metastatic triple-negative breast cancer. U.S. Food and Drug Administration. Published. 2019. Accessed January 24, 2025. https://www.fda.gov/drugs/drug-approvals-and-databases/fda-approves-atezolizumab-pd-l1-positive-unresectable-locally-advanced-or-metastatic-triple-negative

[11] Roche provides update on Tecentriq US indication for PD-L1-positive, metastatic triple-negative breast cancer. https://www.roche.com/investors/updates/inv-update-2021-08-27. 2021. Accessed 2025 January 24.

[12] FDA grants accelerated approval to pembrolizumab for locally recurrent unresectable or metastatic triple negative breast cancer. U.S. Food and Drug Administration. 2020. Accessed February 7, 2025. https://www.fda.gov/drugs/resources-information-approved-drugs/fda-grants-accelerated-approval-pembrolizumab-locally-recurrent-unresectable-or-metastatic-triple

[13] FDA approves pembrolizumab for high-risk early-stage triple-negative breast cancer. U.S. Food and Drug Administration. 2021. Accessed January 24, 2025. https://www.fda.gov/drugs/resources-information-approved-drugs/fda-approves-pembrolizumab-high-risk-early-stage-triple-negative-breast-cancer

[14] National Comprehensive Cancer Network. NCCN Clinical Practice Guidelines in Oncology (NCCN Guidelines®): Breast Cancer Version 3. 2025. https://NCCN.org

[15] Cortes J, Rugo HS, Cescon DW, Im S-A, Yusof MM, Gallardo C, et al. Pembrolizumab plus chemotherapy in advanced triple-negative breast cancer. N Engl J Med. 2022;387(3):217–26. doi: 10.1056/NEJMoa2202809 35857659

[16] Schmid P, Cortes J, Dent R, McArthur H, Pusztai L, Kümmel S, et al. Overall survival with pembrolizumab in early-stage triple-negative breast cancer. N Engl J Med. 2024;391(21):1981–91. doi: 10.1056/NEJMoa2409932 39282906

[17] Ahn JC, Lauzon M, Luu M, Noureddin M, Ayoub W, Kuo A, et al. Racial and ethnic disparities in early treatment with immunotherapy for advanced HCC in the United States. Hepatology. 2022;76(6):1649–59. doi: 10.1002/hep.32527 35429171

[18] Al-Qurayshi Z, Crowther JE, Hamner JB, Ducoin C, Killackey MT, Kandil E. Disparities of immunotherapy utilization in patients with stage III cutaneous melanoma: A national perspective. Anticancer Res. 2018;38(5):2897–901. doi: 10.21873/anticanres.12536 29715114

[19] Haque W, Verma V, Butler EB, Teh BS. Racial and socioeconomic disparities in the delivery of immunotherapy for metastatic melanoma in the United States. J Immunother. 2019;42(6):228–35. doi: 10.1097/CJI.0000000000000264 30985445

[20] Hennink M, Kaiser BN. Sample sizes for saturation in qualitative research: A systematic review of empirical tests. Soc Sci Med. 2022;292:114523. doi: 10.1016/j.socscimed.2021.114523 34785096

[21] Flanigan TS, McFarlane E, Cook S. Conducting survey research among physicians and other medical professionals – a review of current literature. In: Paper presented at: American Statistical Association (ASA): Proceedings of the Section on Survey Research Methods. Alexandria, VA, 2008.

[22] Damschroder LJ, Aron DC, Keith RE, Kirsh SR, Alexander JA, Lowery JC. Fostering implementation of health services research findings into practice: A consolidated framework for advancing implementation science. Implement Sci. 2009;4:50. doi: 10.1186/1748-5908-4-50 19664226 PMC2736161

[23] Tong A, Sainsbury P, Craig J. Consolidated criteria for reporting qualitative research (COREQ): A 32-item checklist for interviews and focus groups. Int J Qual Health Care. 2007;19(6):349–57. doi: 10.1093/intqhc/mzm042 17872937

[24] Valenti RB. Chemotherapy education for patients with cancer: A literature review. Clin J Oncol Nurs. 2014;18(6):637–40. doi: 10.1188/14.CJON.637-640 25427698

[25] Licata L, Dieci MV, De Angelis C, Marchiò C, Miglietta F, Cortesi L, et al. Navigating practical challenges in immunotherapy for metastatic triple negative breast cancer. Cancer Treat Rev. 2024;128:102762. doi: 10.1016/j.ctrv.2024.102762 38776613

[26] Yeung S-CJ, Qdaisat A, Chaftari P, Lipe D, Merlin J, Rajha E, et al. Diagnosis and management of immune-related adverse effects of immune checkpoint therapy in the emergency department. J Am Coll Emerg Physicians Open. 2020;1(6):1637–59. doi: 10.1002/emp2.12209 33392573 PMC7771833

[27] Puzanov I, Diab A, Abdallah K, Bingham CO, Brogdon C, Dadu R, et al. Managing toxicities associated with immune checkpoint inhibitors: consensus recommendations from the Society for Immunotherapy of Cancer (SITC) Toxicity Management Working Group. J Immunother Cancer. 2017;5(1):95. doi: 10.1186/s40425-017-0300-z 29162153 PMC5697162

[28] Covvey JR, Kamal KM, Gorse EE, Mehta Z, Dhumal T, Heidari E, et al. Barriers and facilitators to shared decision-making in oncology: A systematic review of the literature. Support Care Cancer. 2019;27(5):1613–37. doi: 10.1007/s00520-019-04675-7 30737578

[29] New York State Department of Health. New York State Medicaid Update - November 2022 Volume 38 - Number 13. 2022. https://www.health.ny.gov/health_care/medicaid/program/update/2022/no13_2022-11.htm

[30] NCI-Designated Cancer Centers. National Cancer Institute. 2024. Accessed January 22, 2025. https://www.cancer.gov/research/infrastructure/cancer-centers

[31] SB-987 California Cancer Care Equity Act. California Legislative Information. 2022. Accessed January 22, 2025. https://leginfo.legislature.ca.gov/faces/billTextClient.xhtml?bill_id=202120220SB987

[32] CMS Interoperability and Prior Authorization Final Rule CMS-0057-F. U.S. Centers for Medicare & Medicaid Services. 2024. Accessed January 22, 2025. https://www.cms.gov/newsroom/fact-sheets/cms-interoperability-and-prior-authorization-final-rule-cms-0057-f

[33] Emens LA, Adams S, Cimino-Mathews A. Society for Immunotherapy of Cancer (SITC) clinical practice guideline on immunotherapy for the treatment of breast cancer. J Immunother Cancer. 2021;9(8):e002597. doi: 10.1136/jitc-2021-002597 PMC8365813 34389617

[34] National Comprehensive Cancer Network. NCCN Clinical Practice Guidelines in Oncology (NCCN Guidelines®): Distress Management Version 1. 2025. https://NCCN.org

[35] Roberts WL, Soulos PR, Herrin J, Pollack CE, Genao I, Yu JB, et al. Racial and ethnic diversity of the oncology workforce: Projections from 2020 to 2060. JCO Oncol Pract. 2025;21(12):1877–85. doi: 10.1200/OP-25-00876 41074755

[36] Towle E. Demographics of the US oncology workforce. J Oncol Pract. 2016;12(2):99. doi: 10.1200/JOP.2015.010124 26869643

[37] Kegler MC, Liang S, Weiner BJ, Tu SP, Friedman DB, Glenn BA, et al. Measuring constructs of the consolidated framework for implementation research in the context of increasing colorectal cancer screening in federally qualified health center. Health Serv Res. 2018;53(6):4178–203. doi: 10.1111/1475-6773.13035 30260471 PMC6232399

[38] Fernandez ME, Ten Hoor GA, van Lieshout S, Rodriguez SA, Beidas RS, Parcel G, et al. Implementation mapping: Using Intervention mapping to develop implementation strategies. Front Public Health. 2019;7:158. doi: 10.3389/fpubh.2019.00158 31275915 PMC6592155

[39] Reuter K, Genao K, Callanan EM, Cannone DE, Giardina E-G, Rollman BL, et al. Increasing uptake of depression screening and treatment guidelines in cardiac patients: A behavioral and implementation science approach to developing a theory-informed, multilevel implementation strategy. Circ Cardiovasc Qual Outcomes. 2022;15(11):e009338. doi: 10.1161/CIRCOUTCOMES.122.009338 36378766 PMC9909565

